# A Machine Learning Approach Using Survival Statistics to Predict Graft Survival in Kidney Transplant Recipients: A Multicenter Cohort Study

**DOI:** 10.1038/s41598-017-08008-8

**Published:** 2017-08-21

**Authors:** Kyung Don Yoo, Junhyug Noh, Hajeong Lee, Dong Ki Kim, Chun Soo Lim, Young Hoon Kim, Jung Pyo Lee, Gunhee Kim, Yon Su Kim

**Affiliations:** 1 Department of Internal Medicine, Dongguk University College of Medicine, Gyeongju, Korea; 2 Department of Computer Science and Engineering, College of Engineering, Seoul National University, Seoul, Korea; 3 Department of Internal Medicine, Seoul National University College of Medicine, Seoul, Korea; 4 Department of Internal Medicine, Seoul National University Boramae Medical Center, Seoul, Korea; 5 Department of Surgery, College of Medicine, Ulsan University, Asan Medical Center, Seoul, Korea

## Abstract

Accurate prediction of graft survival after kidney transplant is limited by the complexity and heterogeneity of risk factors influencing allograft survival. In this study, we applied machine learning methods, in combination with survival statistics, to build new prediction models of graft survival that included immunological factors, as well as known recipient and donor variables. Graft survival was estimated from a retrospective analysis of the data from a multicenter cohort of 3,117 kidney transplant recipients. We evaluated the predictive power of ensemble learning algorithms (survival decision tree, bagging, random forest, and ridge and lasso) and compared outcomes to those of conventional models (decision tree and Cox regression). Using a conventional decision tree model, the 3-month serum creatinine level post-transplant (cut-off, 1.65 mg/dl) predicted a graft failure rate of 77.8% (index of concordance, 0.71). Using a survival decision tree model increased the index of concordance to 0.80, with the episode of acute rejection during the first year post-transplant being associated with a 4.27-fold increase in the risk of graft failure. Our study revealed that early acute rejection in the first year is associated with a substantially increased risk of graft failure. Machine learning methods may provide versatile and feasible tools for forecasting graft survival.

## Introduction

Kidney transplant (KT) is well recognized as being the best treatment option for patients with end-stage renal disease^1–3^. However, due to the many factors that influence graft survival, accurate prediction of transplant outcomes using standard statistical modelling is difficult^4^. New approaches, such as the use of data mining methods, could improve the precision and accuracy of predicting outcomes of organ transplant by taking into account numerous factors, as well as the complex interactions among these factors. Machine learning, which is the main technical basis for data mining, provides a methodology to extract information from the raw data in medical records^5, 6^. Machine learning has been used to predict graft survival for kidney^7^, liver^8–10^, and heart-lung^11, 12^ transplants. However, as factors that influence the outcomes can vary widely among different organ systems, there is discrepancy regarding the accuracy and effectiveness of prediction models based on machine learning for different graft types.

In the field of KT, computational forecasting has been used in combination with clinical outcomes to predict chronic allograft rejection^13^ delayed graft function (DGF)^14^, and allograft survival^15–20^. Over the past few decades, progress in immunosuppressive therapy, such as the use of cyclosporine, has definitely reduced the incidence rate of acute rejection of kidney grafts, as well as having improved short-term graft survival^21^. Therefore, interest has now shifted to forecasting the long-term survival of kidney allograft.

Several attempts have been made to use machine learning tools for data mining to predict long-term graft survival. These attempts have included the use of decision tree-based modelling learning^15–17^, artificial neural networks^18^ and Bayesian belief networks^19, 20^. Among these different attempts, a good correlation was identified between graft survival predicted using decision tree modelling and the observed 10-year survival rate calculated from survival data in the United States Renal Data System^15^: r-value of 0.98, with an area under the receiver operating curve (AUROC) of 0.90. In another modelling study, using the data from 1,542 KT recipients in the Australian and New Zealand Dialysis and Transplant Registry, the success or failure of a transplant was predicted with an 85% accuracy using artificial neural networks^18^. Bayesian net classifiers have been shown to predict the success or failure of the transplant with 97% accuracy. However, the accuracy for predicting longer-term graft survival duration was lower at 68%^19^. Unfortunately, current computational predictive models of long-term KT graft survival are limited by multiple factors, including: their sole reliance on pre-transplant factors, without consideration of immunological factors^15–20^; relatively small sample size used to build the models^17–19^; and failure to accurately use censored patient data^15–20^. Moreover, the observation time for existing models has been relatively short, while a sufficiently long observation period would be essential to predict long-term graft survival^15, 16^. Due to insufficient research on survival duration of the transplanted kidney and knowledge of the determinants of long-term graft survival, combined with an inability to prioritize the large number of factors known to influence clinical outcomes after KT, current models have largely focused on predicting the success or failure of the transplant and not of its long-term survival^15–19^. Our aim in this study was to use representative data from a Korean population^22, 23^ to compare data mining methods to standard statistical models to predict long-term graft survival after KT.

## Results

### Baseline characteristics

Among the 3,117 KT recipients enrolled in our study, graft failure (GF) occurred in 304 (9.8%) over a mean follow-up period of 85.2 months. Baseline characteristics for patients who experienced GF, compared to those without GF, are summarized in Tables 1 and 2. Among the 304 cases of GF, 201 (66.1%) were male. Compared to the non-GF group, patients who experienced GF also exhibited a higher prevalence of the following pre-transplant risk factors: smoking, a history of viral hepatitis B and C and ischemic heart disease. Other relevant factors were comparable between the two groups, including: pre-transplant dialysis modality, recipients’ age and comorbidities, including diabetes, hypertension, and cerebral and peripheral vascular disease (Table 1).

**Table 1:** Baseline demographic, immunologic characteristics and treatment- associated factors according to graft survival in kidney transplants recipients (KTRs).

	Graft failure (n = 304, 9.8%)	No graft failure (n = 2813, 90.2%)	*P*	Number of missing value (%)
Demographics – Recipients				
Age (years)	41.4 ± 12.1	42.1 ± 11.5	0.332	0 (0.0)
Male gender	201 (66.1)	1655 (58.8)	0.014	0 (0.0)
Body mass index (kg/m^2^)	22.6 ± 3.2	22.3 ± 3.1	0.172	132 (4.2)
Cause of ESRD			<0.001	168 (5.3)
Diabetes	40 (13.5)	355 (13.4)		
Hypertension	13 (4.4)	202 (7.6)		
GN	54 (18.2)	631 (23.8)		
Other	38 (12.8)	472 (17.8)		
Unknown	151 (51.0)	993 (37.4)		
Pre-transplant RRT modality			0.055	1233 (39.5)
Preemptive transplant	17 (6.7)	195 (12.0)		
Hemodialysis	186 (73.2)	1117 (68.6)		
Peritoneal dialysis	39 (15.4)	264 (16.2)		
ABO type			0.206	40 (1.2)
A	133 (43.8)	1056 (37.5)		
B	65 (21.4)	678 (24.1)		
O	35 (11.5)	341 (12.1)		
AB	71 (23.4)	472 (24.7)		
ABOi transplant	25 (8.4)	199 (7.2)	0.476	67 (2.1)
Pre-transplant comorbities				
Smoking status				
Never	234 (77.0)	2205 (78.4)	0.001	1 (0.03)
Former	19 (6.2)	305 (10.8)		
Current	51 (16.8)	302 (10.7)		
Diabetes mellitus	53 (17.4)	480 (17.1)	0.873	1 (0.03)
Hypertension	256 (84.2)	2369 (84.3)	0.976	2 (0.06)
HBsAg positive	25 (8.2)	162 (5.8)	0.003	0 (0.0)
HCV Ab positive	23 (7.5)	89 (3.3)	<0.001	0 (0.0)
Ischemic heart disease	19 (6.2)	108 (3.8)	0.044	1 (0.03)
Cerebral vascular disease	7 (2.3)	77 (2.7)	0.807	1 (0.03)
Peripheral vascular disease	2 (0.7)	12 (0.4)	0.567	1 (0.03)
Immunologic factors				
Donor specific antibody	1 (0.2)	22 (0.7)	0.884	2526 (81.0)
High PRA I titer (>50%)	1 (0.2)	66 (2.3)	0.021	525 (16.8)
High PRA II titer (>50%)	2 (0.2)	38 (1.3)	0.308	635 (20.3)
HLA cross mismatch (+)	6 (1.9)	94 (3.3)	0.509	2 (0.06)
HLA-A mismatch			0.072	105 (3.3)
0	68 (23.1)	796 (29.3)		
1	178 (60.3)	1483 (54.6)		
2	49 (16.6)	438 (16.1)		
HLA-B mismatch			0.001	104 (3.3)
0	34 (11.5)	448 (16.5)		
1	130 (44.1)	1349 (49.6)		
2	131 (44.4)	921 (33.9)		
HLA-DR mismatch			0.024	105 (3.3)
0	45 (15.5)	606 (22.3)		
1	173 (59.7)	1452 (53.4)		
2	72 (24.8)	662 (24.3)		
Treatment factors				
CNIs			<0.001	1 (0.03)
Cyclosporine A	191 (62.8)	1312 (46.7)		
Tacrolimus	112 (36.8)	1497 (53.2)		

Abbreviations: ESRD, end-stage renal disease; GN, glomerular nephritis; RRT, renal replacement therapy; ABOi, ABO incompatible; HBsAg, Hepatitis B virus surface antigen; HCV Ab, hepatitis C virus antibody; CMV IgG, cytomegalovirus immunoglobulin G; PRA, panel reactive antibody; HLA, human leukocyte antigen.

**Table 2:** Baseline characteristics of kidney donors and post-transplant findings according to graft survival in kidney transplants recipients (KTRs).

	Graft failure (n = 304, 9.8%)	No graft failure (n = 2813, 90.2%)	*P*	Number of missing value (%)
Demographics – Donors				
Age (years)	38.4 ± 12.2	39.4 ± 12.1	0.194	96 (3.0)
Male gender	170 (56.9)	1559 (56.9)	0.983	79 (2.5)
Serum creatinine (mg/dL)	1.0 ± 0.6	0.9 ± 0.5	0.039	740 (23.7)
Body mass index (kg/m^2^)	23.3 ± 3.4	23.6 ± 3.3	0.271	402 (12.9)
Donor relation			0.001	38 (1.2)
Living related	128 (42.4)	1475 (53.1)		
Living unrelated	82 (27.2)	660 (23.8)		
Deceased	92 (30.5)	642 (23.1)		
Donor CMV IgG	154 (51.3)	1820 (66.7)	0.001	87 (2.7)
Post-transplant laboratory values at 3 month				
Serum creatinine (mg/dL)	1.7 ± 0.7	1.2 ± 0.5	<0.001	187 (6.0)
Serum Na (mg/dL)	138.8 ± 3.7	140.3 ± 2.8	<0.001	725 (23.2)
Serum total CO_2_ (mg/dL)	23.9 ± 3.4	24.9 ± 3.1	0.001	759 (24.3)
Serum anion gap (mg/dL)	9.5 ± 3.5	9.5 ± 2.6	0.932	766 (24.5)
Post-transplant observational findings				
Infection episode within 1year	54 (17.8)	373 (13.3)	0.031	6 (0.19)
Rejection episode within 1year	73 (24.0)	332 (11.8)	<0.001	0 (0.0)
Total observation period (month)	67.4 ± 51.9	87.9 ± 54.9	<0.001	

Abbreviations: ESRD, end-stage renal disease; GN, glomerular nephritis; RRT, renal replacement therapy; ABOi, ABO incompatible; HBsAg, Hepatitis B virus surface antigen; HCV Ab, hepatitis C virus antibody; CMV IgG, cytomegalovirus immunoglobulin G; PRA, panel reactive antibody; HLA, human leukocyte antigen.

With regard to donor characteristics, 30.5% of patients in the GF group had received a deceased donor transplant, with 53.1% of patients in the non-GF group receiving a living donor transplant. No between-group differences were identified in terms of donor age, sex, kidney function at the time of donation, body mass index, and cytomegalovirus IgG titer (Table 2).

In terms of immunological profiles, HLA-B and donor-recipient (DR) mismatch were the only differences identified between the two groups. Cyclosporine A was more frequently used as the maintenance immunosuppressant therapy among patients with GF, compared to those without GF. Moreover, patients with GF experienced more frequent episodes of infection during the first year post-KT. Laboratory findings at 3 months post-KT were significantly different between the two groups, with higher serum creatinine and lower serum sodium and total CO_2_ levels among patients who experienced GF than among those who did not (Table 2).

### Conventional decision tree modelling of the 10-year graft survival prediction

**Figure 1:**
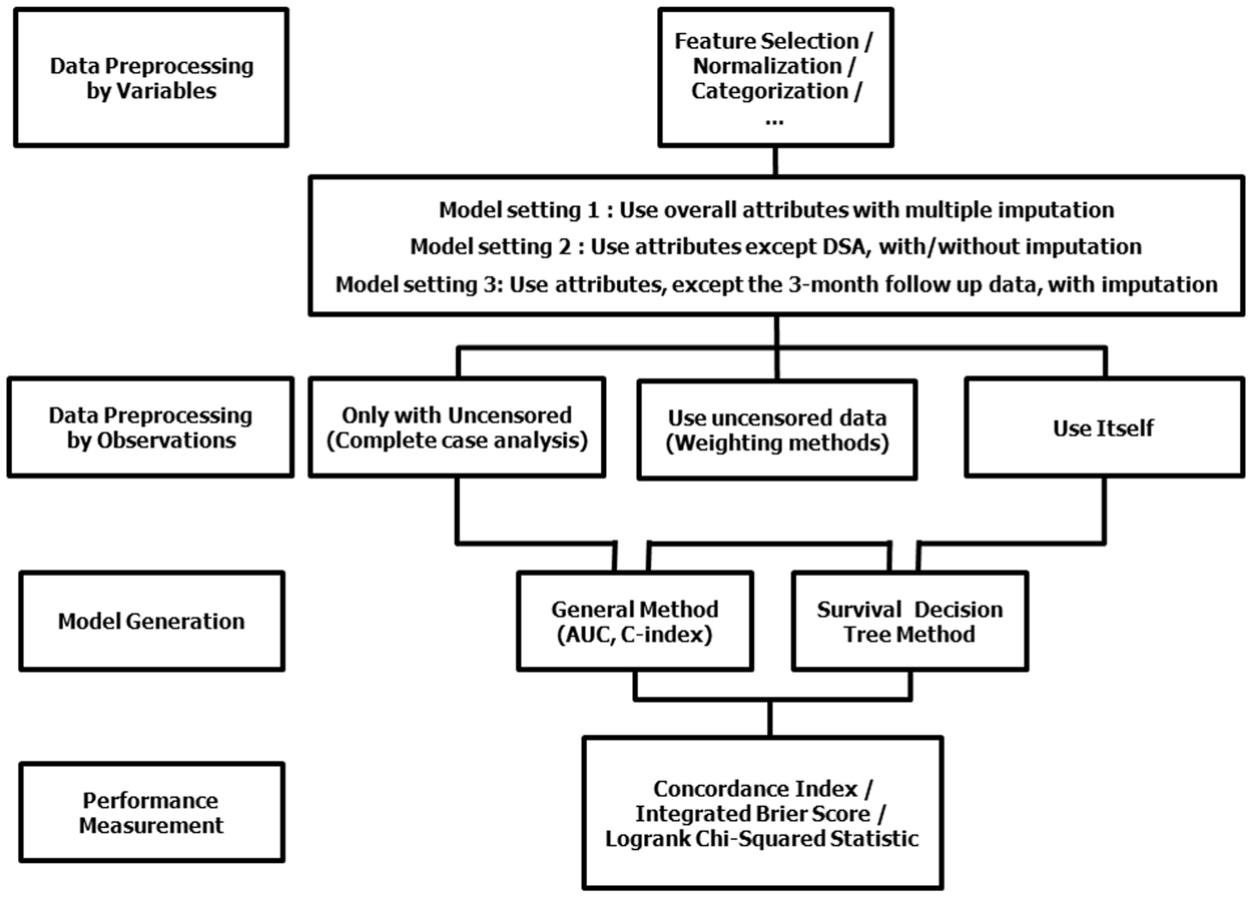
Model structure.

When estimating the survival prediction at N years post-transplant, distortion can be introduced in the estimate when using data from people who have not been followed-up for N years. We tried to overcome the disadvantages of retrospective medical records, including the issue of missing data, with various methods, such as multiple imputation methods (Fig. 1. Setting 1).

In this process, we identified donor-specific antibody (DSA) as a being an important issue to consider. As shown in several retrospective studies using the same dataset, the proportion of DSA-positive patients in our study was very low. We assumed this low prevalence of DSA-positive patients (n = 23) was because these cases did not proceed to transplantation or that a positive DSA was not detected prior to the formal implementation of testing after 2008 (Table 1). The distribution of DSA negative-to-positive patients in our study group was 550:23. Moreover, DSA was not an issue in 2,535 cases. Considering that formal testing was implemented only after 2008, we constructed two different models, one that included DSA (setting 1) and one that did not include DSA (setting 2).

**Figure 2:**
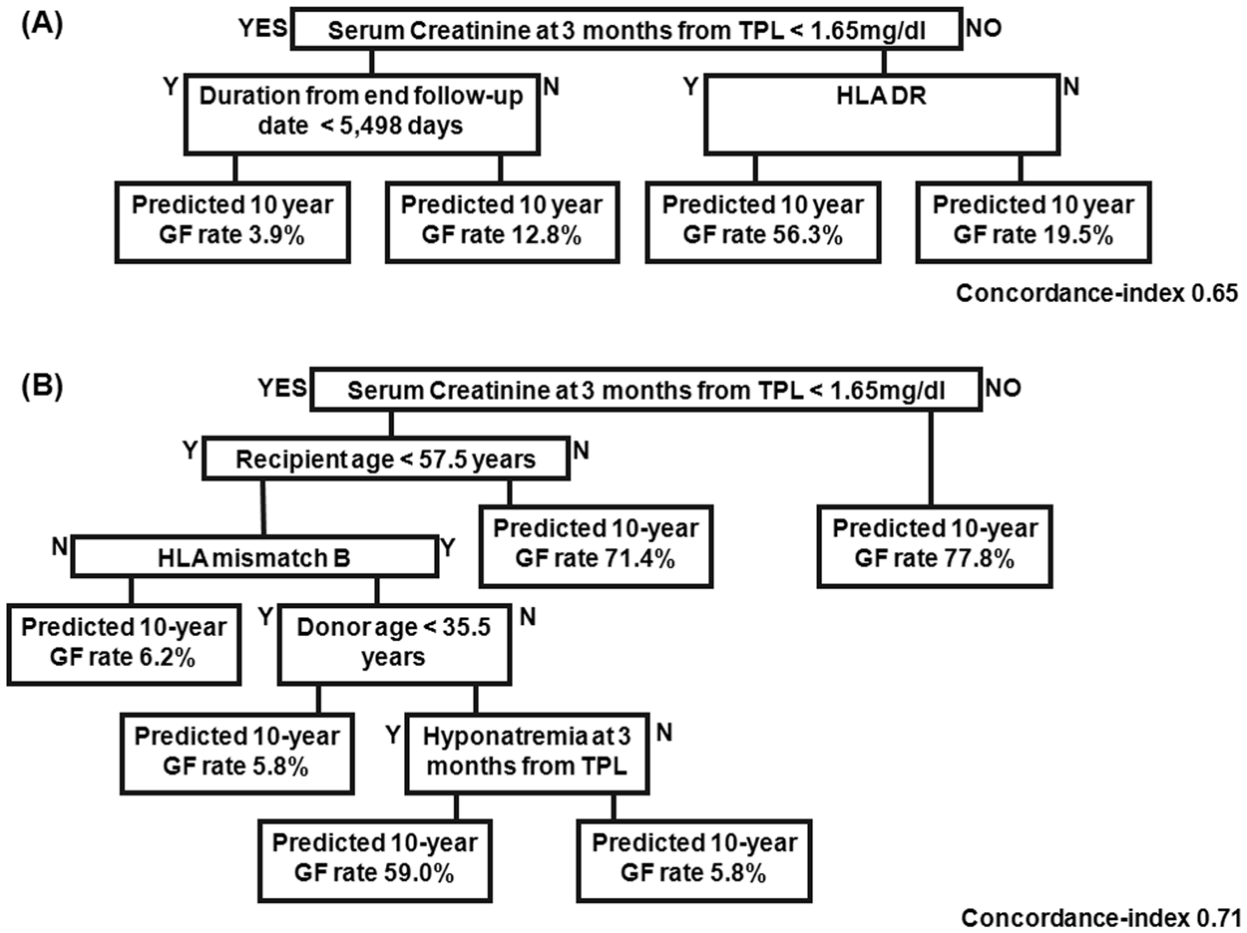
The 10-year graft failure prediction using a decision tree model. Decision tree for the training, test and validation data set, after stratified sampling, with ‘Y’ indicating a positive conclusion and ‘N’ a negative conclusion. The 10-year graft failure rate is reported as a percentage. (**A**) Model setting 1: Use overall attributes with multiple imputation. (**B**) Model setting 2: Use attributes except DSA, with/without imputation

Parameters used in our predictive model is summarized in Table 3 including: the method of imputation, test ratio, validation method, validation ratio, N folds, training and test performance of the classifiers on the test set, and validation of the mixed dataset in conventional decision tree using different model setting. The performance of the classifier, with the associated index of concordance, is also reported in Table 3. The routines used to implement conventional classification and regression trees (CART) are shown in Fig. 2 ^24, 25^.

**Table 3:** Performance of the prediction model by conventional decision tree using different model setting.

Setting	Imputation method	Use One	Weight method	Validation method	Validation ratio	N folds	Train set size	Test set size	Parameters	Train Performance	Test Performance
1	MICE/CART	T	Zupan	Cross-validation		5	993	331	cp = −1/maxdepth = 14	0.62	0.64
1	MICE/CART	T	nothing	Cross-validation		5	993	331	cp = 0.002/maxdepth = 6	0.78	0.62
1	MICE/CART	F	nothing	Cross-validation		5	855	285	cp = −1/maxdepth = 2	0.70	0.61
1	MICE/CART	T	Zupan	One validation	0.285		993	331	cp = −1/maxdepth = 14	0.62	0.59
1	MICE/CART	T	nothing	One validation	0.285		993	331	cp = −1/maxdepth = 2	0.72	0.59
1	MICE/CART	F	Zupan	One validation	0.285		855	285	cp = −1/maxdepth = 12	0.61	0.52
1	MICE/CART	F	Zupan	Cross-validation		5	855	285	cp = −1/maxdepth = 20	0.62	0.48
1	MICE/CART	F	nothing	One validation	0.285		855	285	cp = −1/maxdepth = 8	0.91	0.46
2	nothing	T	nothing	One validation	0.285		288	96	cp = 0.002/maxdepth = 6	0.83	0.71
2	nothing	T	Zupan	Cross-validation		5	288	96	cp = −1/maxdepth = 20	0.63	0.71
2	nothing	F	Zupan	Cross-validation		5	231	77	cp = −1/maxdepth = 6	0.60	0.70
2	MICE/CART	T	Zupan	Cross-validation		5	993	331	cp = −1/maxdepth = 24	0.64	0.67
2	nothing	T	nothing	Cross-validation		5	288	96	cp = −1/maxdepth = 8	0.92	0.66
2	nothing	T	Zupan	One validation	0.285		288	96	cp = −1/maxdepth = 10	0.61	0.65
2	MICE/CART	T	nothing	Cross-validation		5	993	331	cp = 0.008/maxdepth = 10	0.73	0.64
2	MICE/CART	T	nothing	One validation	0.285		993	331	cp = −1/maxdepth = 6	0.83	0.62
2	nothing	F	nothing	One validation	0.285		231	77	cp = 0.002/maxdepth = 4	0.88	0.62
2	MICE/CART	F	Zupan	One validation	0.285		855	285	cp = −1/maxdepth = 16	0.62	0.55
2	MICE/CART	F	Zupan	Cross-validation	—	5	855	285	cp = −1/maxdepth = 18	0.61	0.55
2	MICE/CART	T	Zupan	One validation	0.285		993	331	cp = −1/maxdepth = 16	0.63	0.54
2	nothing	F	nothing	Cross-validation		5	231	77	cp = −1/maxdepth = 2	0.79	0.53
2	nothing	F	Zupan	One validation	0.285		231	77	cp = −1/maxdepth = 6	0.61	0.53
2	MICE/CART	F	nothing	Cross-validation		5	855	285	cp = −1/maxdepth = 4	0.82	0.49
2	MICE/CART	F	nothing	One validation	0.285		855	285	cp = −1/maxdepth = 6	0.92	0.47
3	MICE/CART	T	nothing	Cross-validation		5	993	331	cp = −1/maxdepth = 6	0.86	0.62
3	MICE/CART	T	Zupan	Cross-validation		5	993	331	cp = −1/maxdepth = 16	0.63	0.61
3	MICE/CART	T	nothing	One validation	0.285		993	331	cp = −1/maxdepth = 6	0.83	0.61
3	MICE/CART	T	Zupan	One validation	0.285		993	331	cp = −1/maxdepth = 14	0.64	0.60
3	MICE/CART	F	Zupan	One validation	0.285		855	285	cp = −1/maxdepth = 16	0.61	0.58
3	MICE/CART	F	nothing	One validation	0.285		855	285	cp = −1/maxdepth = 4	0.76	0.53
3	MICE/CART	F	nothing	Cross-validation		5	855	285	cp = −1/maxdepth = 6	0.83	0.51
3	MICE/CART	F	Zupan	Cross-validation		5	855	285	cp = −1/maxdepth = 26	0.62	0.48

*Test performance were presented as concordance index for time to graft failure data. Model setting 1: Use overall attributes with multiple imputation. Model setting 2: Use attributes except DSA, with/without imputation. Model setting 3: Use attributes except 3 month follow up data, with imputation. Test ratio fixed at 0.3. Use One False (F) is used if the follow-up period is shorter than the period to be predicted in the classification; these cases were excluded from the training process. Use One True (T) is used as a positive example if the patient has experienced a graft failure even though the follow-up period is short. Weight method by Zupan *et al*. is used when the follow-up period is short, with both positive and negative examples used, but with different weights^6^.

The binary classification tree derived from the dataset by conventional methods is shown in Fig. 2, with the index of concordance ranging between 0.65 to 0.71. In the decision tree, internal nodes represent attributes, with leaf nodes clarifying the relative frequency of graft failure being equal to ‘1’.

Among various conventional decision tree (DT) models, the 3-month serum creatinine level post-transplant was identified at the first decision node, being the most important risk factor of GF (predicted 10-year GF rate, 77.8%; Fig. 2(B), setting 2, index of concordance 0.71). The age of the recipient at the time of transplantation was selected at the next node in the group of patients with a 3-month serum creatinine level <1.65 mg/dL, with a predicted 10-year GF rate of 71.4% for patients without HLA mismatch and ≥57.5 years of age, with the GF rate decreasing to 6.2% for patients <57.5 years of age. For patients <57.5 years of age, hyponatremia at 3 months post-KT and the age of the donor were selected at the next node, in the presence of a positive B HLA mismatch.

In setting the process for 10 year graft survival prediction, we firstly applied a conventional decision-tree model from 1 to 10 years after KT, and additionally verified applying weighting method (Table 4)^6^. However, these models were problematic to use with participants with a shorter follow-up period. Therefore, we used a different predictive approach based on survival statics.

**Table 4:** Comparison of AUC values between baseline model (only with uncensored data) and weighting model in decision-tree modeling for classification problem.

Period after KT for prediction (year)	1 yr	2 yr	3 yr	4 yr	5 yr	6 yr	7 yr	8 yr	9 yr	10 yr
Baseline model AUC^a,b^ (complete case analysis)	0.9728	0.8856	0.7916	0.7523	0.7121	0.7164	0.6707	0.6859	0.6714	0.6583
Weighting model AUC^a,b^	0.9754	0.8845	0.7958	0.7389	0.7013	0.720	0.7107	0.7150	0.7013	0.7066

^a^Setting 1: Use overall attributes with multiple imputation, ^b^Instance weighting methods by Zupan *et al*.^6^; yr, year.

### Decision tree modelling using survival statics, with comparisons to previous conventional models

The following machine learning modelling algorithms were applied: survival decision tree, bagging, random forest, and ridge and lasso. These models use the survival analysis statistic^26^, rather than the Gini index or entropy index for the split rule used in conventional tree algorithms. The parameters of the survival model, including the index of concordance, were presented in Table 5. The survival tree algorithm performed better than conventional tree modelling, with an index of concordance of 0.80 compared to 0.71, respectively. GF in survival tree models was predicted as a survival hazard ratio (HR), with overall graft survival within the study group used as the reference value.

**Table 5:** Performance of the prediction model by survival hazard ratio decision tree using different model setting.

Setting	Imputation method	Use One	Weight method	Validation method	Validation ratio	N folds	Train set size	Test set size	Parameters	Training Performance	Test Performance
1	MICE/CART	F	nothing	Cross-validation		5	2796	932	cp = 0.01/maxdepth = 6	0.73	0.71
1	MICE/CART	T	nothing	Cross-validation		5	2796	932	cp = 0.01/maxdepth = 6	0.71	0.68
1	MICE/CART	T	nothing	One validation	0.285		2796	932	cp = −1/maxdepth = 2	0.72	0.67
1	MICE/CART	F	nothing	One validation	0.285		2796	932	cp = 0.004/maxdepth = 14	0.92	0.59
2	nothing	F	nothing	One validation	0.285		930	310	cp = 0.028/maxdepth = 4	0.78	0.80
2	MICE/CART	F	nothing	Cross-validation		5	2796	932	cp = 0.008/maxdepth = 4	0.74	0.69
2	MICE/CART	F	nothing	One validation	0.285		2796	932	cp = 0.01/maxdepth = 6	0.77	0.68
2	MICE/CART	T	nothing	Cross-validation		5	2796	932	cp = 0.008/maxdepth = 6	0.71	0.68
2	MICE/CART	T	nothing	One validation	0.285		2796	932	cp = −1/maxdepth = 2	0.72	0.67
2	nothing	F	nothing	Cross-validation		5	930	310	cp = −1/maxdepth = 12	0.94	0.62
2	nothing	T	nothing	Cross-validation		5	930	310	cp = 0.002/maxdepth = 12	0.94	0.61
2	nothing	T	nothing	One validation	0.285		930	310	cp = 0.02/maxdepth = 8	0.91	0.60
3	MICE/CART	F	nothing	Cross-validation		5	2796	932	cp = 0.012/maxdepth = 4	0.72	0.68
3	MICE/CART	T	nothing	Cross-validation		5	2796	932	cp = 0.014/maxdepth = 4	0.70	0.65
3	MICE/CART	F	nothing	One validation	0.285		2796	932	cp = 0.01/maxdepth = 6	0.74	0.61
3	MICE/CART	T	nothing	One validation	0.285		2796	932	cp = 0.014/maxdepth = 4	0.72	0.58

*Test performance were presented as concordance index for time to graft failure data. Model setting 1: Use overall attributes with multiple imputation. Model setting 2: Use attributes except DSA, with/without imputation. Model setting 3: Use attributes except 3 month follow up data, with imputation. Test ratio fix 0.3. Use One False (F) is used if the follow-up period is shorter than the period to be predicted in the classification; these cases were excluded from the training process. Use One True (T) is used as a positive example if the patient has experienced a graft failure even though the follow-up period is short. Weight method by Zupan *et al*. is used when the follow-up period is short, with both positive and negative examples used, but with different weights^6^.

**Figure 3:**
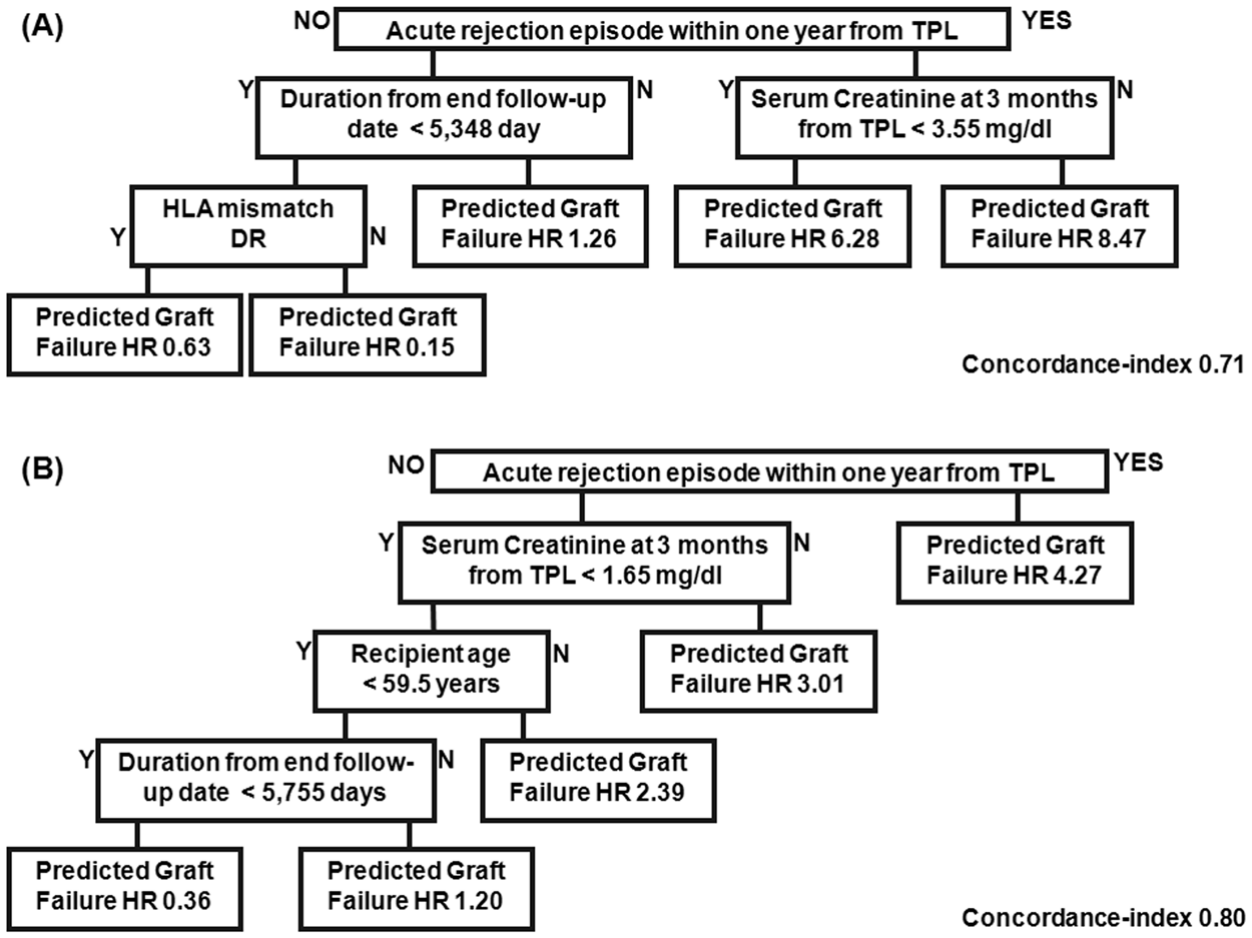
The graft survival prediction tree using survival hazard ratio modelling. Decision tree using the training, test and validation data set, after stratified sampling using survival statics, with ‘Y’ indicating a positive conclusion and ‘N’ a negative conclusion. The graft failure risk is presented as a survival hazard ratio (HR). (**A**) Model setting 1: Use overall attributes with multiple imputation. (**B**) Model setting 2: Use attributes except DSA, with/without imputation

The survival tree algorithm identified an episode of acute rejection within the first year after KT to be the most deterministic node for predicting GF (Fig. 3(B), setting 2), resulting in a 4.27-fold increase in the risk of GF compared to the overall GF rate for the study group. The 3-month serum creatinine level and the age of the recipient were significantly associated with allograft survival. Among patients with normal serum creatinine level (<1.65 mg/dL) who did not experience an episode of graft rejection in the first year post-transplant, recipients’ age was the next predictive node of graft survival. Specifically, among patients who did not experience an episode of rejection over the first years after KT, a 3-month serum creatinine level >1.65 mg/dL was predictive of GF (HR, 3:01). Age was an independent risk factor of GF, with a HR of GF of 2.39 for patients over the age of 59.5 years, even in the absence of an episode of rejection.

Results for the application of the conventional Cox survival model for setting 1, 2, and 3 are summarized in Table 6. The Cox model performed at an index of concordance of only 0.60–0.63.

**Table 6:** Performance of the prediction model by survival cox regression analysis using different model setting.

Setting	Imputation method	Use One	Weight method	Validation method	Validation ratio	Train set size	Test set size	Parameters	Training Performance	Test Performance
1	MICE/CART	F	nothing	One validation	0.285	2796	932	Nothing	0.78	0.62
1	MICE/CART	F	nothing	One validation	0.285	2796	932	Nothing	0.78	0.62
1	MICE/CART	T	nothing	One validation	0.285	2796	932	Nothing	0.77	0.60
1	MICE/CART	T	nothing	One validation	0.285	2796	932	Nothing	0.77	0.60
2	Nothing	F	nothing	One validation	0.285	930	310	Nothing	0.82	0.65
2	Nothing	F	nothing	One validation	0.285	930	310	Nothing	0.82	0.65
2	Nothing	T	nothing	One validation	0.285	930	310	Nothing	0.81	0.63
2	Nothing	T	nothing	One validation	0.285	930	310	Nothing	0.81	0.63
2	MICE/CART	F	nothing	One validation	0.285	2796	932	Nothing	0.78	0.62
2	MICE/CART	F	nothing	One validation	0.285	2796	932	Nothing	0.78	0.62
2	MICE/CART	T	nothing	One validation	0.285	2796	932	Nothing	0.77	0.60
2	MICE/CART	T	nothing	One validation	0.285	2796	932	Nothing	0.77	0.60
3	MICE/CART	F	nothing	One validation	0.285	2796	932	Nothing	0.79	0.62
3	MICE/CART	F	nothing	One validation	0.285	2796	932	Nothing	0.79	0.62
3	MICE/CART	T	nothing	One validation	0.285	2796	932	Nothing	0.78	0.61
3	MICE/CART	T	nothing	One validation	0.285	2796	932	Nothing	0.78	0.61

*Test performance were presented as concordance index for time to graft failure data. Model setting 1: Use overall attributes with multiple imputation. Model setting 2: Use attributes except DSA, with/without imputation. Model setting 3: Use attributes except 3 month follow up data, with imputation. Test ratio fix 0.3. Use One False (F) is used if the follow-up period is shorter than the period to be predicted in the classification; these cases were excluded from the training process. Use One True (T) is used as a positive example if the patient has experienced a graft failure even though the follow-up period is short. Weight method by Zupan *et al*. is used when the follow-up period is short, with both positive and negative examples used, but with different weights^6^.

## Discussion

Machine learning forms the basis of data mining and provides a robust methodology to extract information from extremely large databases^5, 6^. This methodology is particularly well suited to the field of transplant medicine, where numerous factors influence clinical outcomes^5–7^. Our study provides several major advantages to underline the feasibility of using machine learning approaches to predict graft survival after KT. First, we applied the majority of known machine learning methods (survival decision tree, bagging, random forest, and ridge and lasso) to a large dataset obtained from three centers in Korea, having expertise in KT. Second, we maximized the use of censored patient data, with survival statistics providing meaningful clinical information (Fig. 3)^24–26^. Third, we prioritized factors predictive of allograft survival. Using tree modelling with survival statistics, we identified acute rejection, confirmed by renal biopsy, during the first year after KT as being the greatest risk factor for graft loss, regardless of serum creatinine levels (adjusted HR, 4.27). Among patients who did not experience an episode of acute rejection within the first year after KT, the 3-month level of serum creatinine was the most important risk factor of graft loss (adjusted HR, 3.01; Fig. 3). These predictive factors were identified by considering post-transplant laboratory findings in combination with 33 attributes of the pre-transplant status of recipients and characteristics of donors in the model (Table 5).

Although the association between biopsy-proven acute rejection (BPAR) and early graft loss is well-known^4^, as well as its potential impact on the long-term graft survival, regardless of kidney function, there is controversy as to whether rejection-episode by histologic findings is how much influence it has for graft survival^27–29^. Several studies have evaluated the effect size of rejection episode on long-term graft survival, and the debate has persisted as to whether early (from 3 month to 2 years post-transplant) acute rejection is a cause of long-term graft failure^30–33^. Of note, the most recent data on GF post-transplantation identified an association between acute rejection in the first six months after KT and death-censored GF among 83,008 deceased donor kidney transplant recipients and 48,399 living donor kidney transplant recipients, adjusting for recipient characteristics and the kidney donor profile index (KDPI) for living donors^33^. Therefore, acute rejection in the first six months post-transplant substantially increases the risk of early and late graft loss^33^. Using a time varying Cox regression analysis, Koo *et al*. reported that both early acute rejection and late acute rejection were related to GF in a Korean population^30^. These results are consistent with our findings.

Many studies have investigated the relationship between graft survival and the timing of acute rejection, and it seems clear that late transplant rejection has a deleterious effect on the survival rate of organs^31, 32, 34, 35^. The persisting debate on the effects of early acute rejection on the long-term graft survival may be due to differences in how the rejection episode is defined between studies, as well as differences in the characteristics of the study population. As an example, previous studies that have reported a negative association between early acute rejection and graft loss limited their analysis to a rejection event sustained within the first 3 months post-transplant^34, 35^. In addition, the initial diagnosis of graft rejection was made clinically without confirmation by biopsy^35^. Studies conducted in Korea have defined early acute rejection as an event that occurs up to 1-year post-transplant^30^.

Among patients who did not develop transplant rejection within 1 year in our study group, the level of serum creatinine at 3 months post-transplant was the most important predictor of graft loss, with age of the recipient and length of observation from the time of transplant being additional risk factors (Fig. 3(B)). The predictive value of serum creatinine or estimated glomerular filtration ratio changes for long-term survival of a kidney graft has been debated^36–39^. Recently, using data from the Australia and New Zealand Dialysis and Transplant Registry, Clayton *et al*. identified a 30% decrease in the glomerular filtration rate (GFR) at 1-year post-transplant, from baseline obtained immediately after KT, to be the best predictor of graft survival, in combination with a 2-fold increase in serum creatinine and absolute glomerular filtration rate^40^. Our study does support these findings, with early graft rejection and the 3-month serum creatinine level being predictive of long-term graft survival.

**Figure 4:**
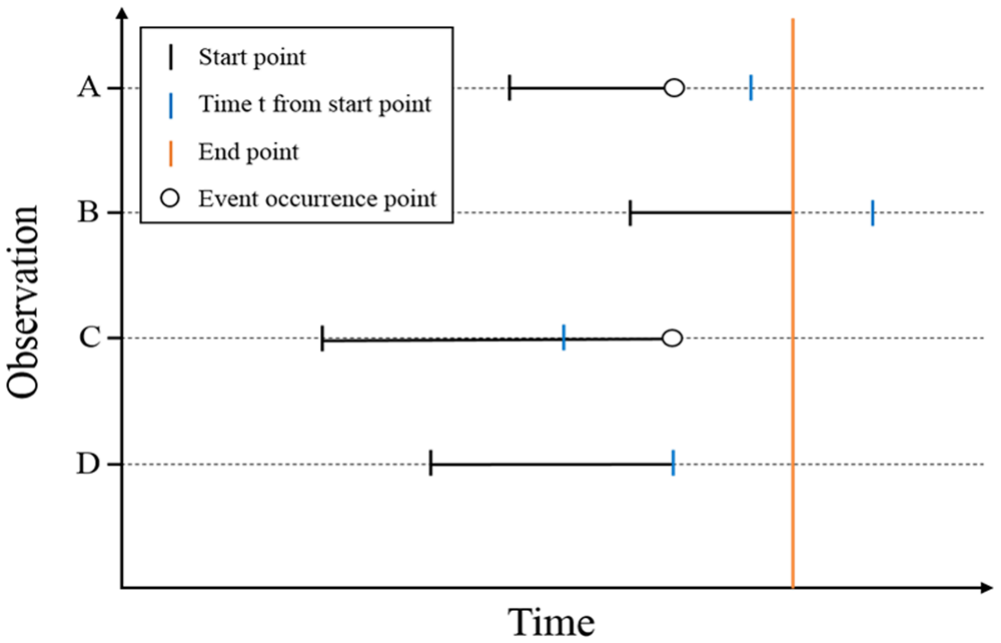
Limitation of conventional prediction model using censored data. The blue line indicates the time point ‘*t*’, from the starting point of observation, for each case.

An important precaution when using a machine learning approach with an observational cohort is the presence of prevalence data obtained from censored observations. Such observational data are not easy to handle, as they do not provide deterministic information to predict final patient outcomes. The panel in Fig. 4 shows an example of censored data. When predictor variables are present from the start point of observation, regression modelling can be accurately applied, such as for observations presented in Fig. 4(A and C), which occurred before the last observation. In contrast, observations (B) and (D) in the same Figure are problematic due to the lack of specific knowledge on when the event will recur in the future. This problem extends to the classification process, with the recurrence of an event after time ‘*t*’, from a certain time point of observation, being predictable for observations in Fig. 4A (event occurs) and C and D (event does not occur), but with the time-point of recurrence being unknown for observation B. Therefore, the presence of censored observations creates regression and classification problems that make accurate prediction difficult^24–26^. To overcome these problems, classification can proceed using censored date, with survival statistics applied to various machine learning techniques with different attribute settings (Fig. 1)^26^. In addition, since graft rejection is a time varying event, there is inevitable limitation to using conventional statistical methods, such as Cox regression. Because of the characteristics of retrospective data, there are time-fixed variables that take into account the baseline characteristics for all covariates, except for the time point of the acute rejection. To address this issue, we implemented the following conditions to our predictive modelling. First, if the follow-up period of the patient was shorter than the period to be predicted in the classification, the patient-specific data was excluded from the training process, identified by the category “Use One: FALSE” in Tables 3, 5 and 6 (condition 1). If an acute rejection event was not identified over a follow-up period >1 year, the rejection event was coded as ‘0’ (condition 1–1). For cases in which a rejection event was observed, the event was coded a ‘0’ if it occurred within the first year post-transplant and a ‘1’ if it occurred at >1 year post-transplant (condition 1–2). All other ‘FALSE’ events were coded as ‘2’ (not treated). In cases in which the patient experienced GF despite a short follow-up period, the event was categorized as “Use One: TRUE” in Tables 3, 5 and 6 (condition 2). If the acute rejection event associated with the ‘TRUE’ graft failure event occurred at >1 year follow-up, the event was coded as ‘0’ (condition 2–1), with the event coded as ‘1’ if the acute rejection occurred at <1 years post-transplant (condition 2–2). All other TRUE events were coded as ‘2’ (not treated).

In our study, we applied these techniques to three types of tree-based models: survival decision tree, bagging and random forest models^41–43^. Survival tree modelling (Table 5) has been shown to have higher predictive power than existing conventional decision tree models, increasing the reliability of identified factors in predicting clinical outcome^26^. Moreover, we showed the comparison between the performance of our decision tree models and a standard Cox regression analysis in Tables 3 and 6. Compared to a Cox regression analysis, decision tree modelling has the advantage of clarifying the classification and prediction processes, represented by the inference rule of the tree structure. Several prior studies have attempted to predict survival of kidney grafts using conventional decision tree models^15–17, 44^. Based on the data of 92,844 KT recipients, Krikov *et al*. predicted the 10-year survival with high accuracy (AUROC, 0.90)^15^. However, the researchers did not identify the factors associated with graft loss. Focusing on recipients of grafts from living donor from a single center having KT experience in Egypt, Fouad *et al*. also compared decision tree classifiers to regression analysis^17^. However, their comparison was limited by insufficient information regarding their model attributes, inclusion of pre-transplant information only and a relatively small study group. In our study, we also tried conventional decision tree modelling to predict the 10-year graft survival with weighting method (Tables 3 and 4). A 3-month serum creatinine level >1.65 mg/dL was associated with a GF rate of 77.8% (Fig. 2). The next most important predictive factor was the age of the recipient at the time of transplant, with age >57.5 years being associated with a 71.5% rate of GF, despite serum creatinine levels <1.65 mg/dL. For recipients <57.5-years-old, a HLA B mismatch was associated with a 10-year GF rate of 6.2%. In this group, donor age was also an important predictor of graft survival, with more favorable outcomes identified when grafts were obtained from young donors. A donor age >35 years was associated with hyponatremia at 3 months post-KT, with a rate of GF as high as 59%. It is well known that the effects of HLA DR and B mismatch on graft outcome are considerable, while an HLA-A mismatch does not produce a significant effect^45, 46^. In our study, the risk of HLA-B mismatch was higher among younger patients compared to that of HLA DR. Further research is needed to clarify biological mechanism of HLA DR mismatch associated graft rejection.

The limitations of our study need to be acknowledged. First, donor and recipients ethnicity was not considered in our models. Previous studies from western countries have included Asians living in western countries. It is likely that environmental differences will influence clinical outcomes for patients of the same ethnicity living in different countries. Moreover, the characteristics of Asian individuals are likely to vary even between middle and eastern regions of Asia (Japan, China, and Korea). Overall, the inclusion of Asian populations has been quite limited in studies of kidney transplantation. In fact, in the landmark trial for transplant outcomes by Wolfe *et al*., only 3.9% of the study sample was Asian^47^. We have tried to overcome these limitations by using a study sample that is representative of Asians who live in Asia, eliminating effects of environmental, socio-cultural and racial differences. The second limitation is the high proportion of missing values of donor-specific antibody (DSA) data, even though we applied different model settings to address this issue (Fig. 1). Moreno-Gonzalez *et al*. presented an excellent retrospective study of an allograft prediction model using a combination of alloantibody and one-year surveillance data^48^, which included DSA information at 1 year for 42% of their cases, compared to 18% in our study. Adding this information, biopsy rejection score 1 year post-transplant and DSA, to the model for death–censored graft loss increased the predictability (C statistic = 0.90). Shabir *et al*. provided a more detailed method for predicting outcomes, with external validation against data from a multicenter cohort^49^. This study showed good-to-excellent discrimination for death-censored transplant failure (C statistics, 0.78–0.90) using data at 12-months post-transplant to develop their model. The absence of external validation is a major limitation of our study.

Despite these limitations, our model survival decision tree model improved the accuracy of predicting GF (index of concordance, 0.80) over conventional decision tree and Cox regression models. As such, our model might be clinically relevant to identify patients with a specific group of risk factors associated with graft loss and, thus, enable clinicians to predict outcomes of KT. Moreover, we do provide evidence supporting the application of advanced machine learning techniques, using survival statistics, in the field of KT. Using this approach, we have prioritized risk factors of graft failure, with acute rejection, confirmed by renal biopsy, within 1 year of KT being the most important risk for graft failure. We also emphasized the importance of post-transplant serum creatinine level as a predictive factor. Our findings will assist clinicians in predicting outcomes of KT.

## Methods

### Study participants

Our study cohort included 3,117 adults (>18-years-old) who had undergone KT at three tertiary care hospitals with expertise in KT in Korea, between 1997 and 2012. Our analysis included >50 attributes extracted from 3,117 medical records of recipients and donors, which included complete records for 2,017 recipients. The institutional review board at each hospital approved our methods (Institutional Review Board No. H-1409-086-609) and the study was performed according to the ethical standards of the Helsinki Declaration. This study is a retrospective, non-interventional study, and written informed consent was waived due to the study’s design.

### Attributes used for modelling

The following recipient-related factors were extracted from the medical records for analysis: age; sex; smoking, primary cause of end-stage renal disease (ESRD), body mass index (BMI), ABO type and ABO incompatible transplant; infection history within 1 year after transplant, type of maintenance immunosuppressant (cyclosporine *versus* tacrolimus); and a history of pre-transplant ischemic heart disease, peripheral vascular disease, diabetes mellitus, hypertension, and hepatitis B or C virus infection. Duration of observation from the endpoint of follow-up (23 March 2015) was included as a attribute, measured from the time of transplant to the endpoint of follow-up, which reflects the transplant era.

The following pre-transplant laboratory levels were also obtained for analysis: serum creatinine, hemoglobin, white blood cells (WBC), albumin, cholesterol, low-density lipoprotein (LDL), and high-sensitivity C-reactive protein. The following pre-transplant immunological factors were also included in the analysis: donor specific antibody and panel reactive antibody titer. The following laboratory findings obtained at 3 and 6 months post-transplantation were also included in the analysis: serum creatinine; serum Na; total CO_2;_ calculated anion gap; and blood levels of cyclosporine and tacrolimus. The following donor-related factors were included in the analysis: age, sex, BMI, donor type, cytomegalovirus IgG titer, miss-match of human leukocyte antigen (HLA) A, B and DR, and cross matching results, were reviewed.

Among all factors considered, 33 independent attributes likely to influence graft survival were selected for inclusion in our prediction models, using individual and ensemble learner with multiple imputation (Tables 1 and 2).

### Modelling process

We created training sets for machine learning using 80% of the overall dataset. We ensured a 10-fold cross validation for our training process, with performance of the trained model evaluated using 20% of the overall dataset. We used five different seeds to measure the final performance, with the index of concordance considered to be the main criteria to evaluate model performance. Two widely used individual learning models (Classification and Regression Trees, Logistic Regression)^24, 25^ and ensemble learning models (Bagging and Random Forest)^42, 43^ were implemented.

### Imputation method

Our study included data obtained over an 18-year period, from 1997 to 2012. To overcome the problem of differences in transplant technique across this time period, including differences in variables evaluated pre-transplantation and monitored after transplant, we evaluated multiple data imputation methods, defined in statistical libraries in R, selecting the multivariate imputation by chained equation (MICE) method, with supplemented attributes imputation of missing values^24^. R statistical language (Version R 3.0.2, The Comprehensive R Archive Network: http://cran.r-project.org) and the MICE package were used for imputing missing values for continuous and categorical data (CART, Random Forest, Sample; Random sample from the observed values).

### Individual learners

Decision tree, also called Classification and Regression Trees (CART), is a conceptually simple approach but one that is statistically powerful^25^. As decision trees are more expressive then other models in terms of their classification process, they are easier to implement and interpret than many other machine learning algorithms, as well as being applicable to non-linear datasets. CART formulation forms a binary tree and minimizes the training error in each leaf. CART uses a Gini coefficient to choose the best variable, which estimates the purity of the internal nodes. Tree models represent data by a set of binary decision rules^25^. Logistic regression is based on the logistic function with a linear combination of dependent variables, and is formulated as: π(x) = 1/1 + e − (β X) where βt X = β0 + β1 × 1 + β2 × 2 + …, with π(x) as the probability p(y = 1|X) that the dependent variable (y) is of class 1, given the independent variables(xi)^25^. We also used the generalized linear model (GLM) library in R to fit the logistic regression model^41^. The GLM for baseline modelling used a binomial distribution for the response with a logit link function^41^.

### Ensemble learners

Ensemble methods classify data by combining the results of multiple learners, with the aim of improving the predictive performance of a given statistical learning model or a fitting technique. Bagging and random forest are different ensembling techniques. Bagging is the acronym of bootstrap aggregating^42^, which builds predictive models by using repeated bootstrap samples from training dataset and aggregates those predictors. For aggregation, the average is used for regression model and plurality vote for classification model^42^. We chose CART as a base learner among various algorithms.

The random forest algorithm adds more randomness to bagging. However, using a decision tree as base learner, the random forest algorithm randomly chooses a specific number of attributes at each node and finds the best split among these attributes^43^.

### Module structure

Conventional prediction models are inherently limited by censored data used with individual and ensemble learners (Fig. 4). Typically, this limitation is addressed by omitting these data, which often results in an insufficient follow-up period for prediction modelling. An alternative solution to omission is treating censored data as non-recurring samples (classification) and treating their follow-up time as survival time (regression). However, both of these solutions introduce bias, with this biasing effect being large if the rate of event occurrence is low. To avoid such biasing effects, we included all censored data in our models using survival statistics (Fig. 4)^26^.

### Decision tree using survival statics

A general decision tree was built using a process of recursively finding a split rule, such as the Gini index or entropy index, which lowered the impurity in classified data of patients’ outcome. Survival decision tree uses survival statistics as the split rule criteria, which is formulated as follows: (1) *c*_*i*: the observed event count for observation *i*, (2) *t*_*i*: the observation time for observation *i*, (3) Observed event rate: $\widehat{\lambda }=\frac{\#\,events}{total\,time}=\frac{{\sum }^{}{c}_{i}}{{\sum }^{}{t}_{i}}$, (4) Within node deviance: $D=\frac{1}{N}{\sum }^{}[{c}_{i}\,\mathrm{log}(\frac{{c}_{i}}{\widehat{\lambda }{t}_{i}})-({c}_{i}-\widehat{\lambda }{t}_{i})]$, and (5) Maximize the improvement: ${D}_{parent}-({D}_{left}+{D}_{right})$.

The goal is to maximize the improvement for each split, which is equivalent to minimizing the within-node deviation. Based on this process, we can identify the tree model that maximizes the likelihood of the a specific classification at a specific node and, thus, optimizes the fit of the data^26^.
